# Occurrence of fluke infection in beef cattle around Phayao Lake, Phayao, Thailand

**DOI:** 10.14202/vetworld.2020.334-337

**Published:** 2020-02-20

**Authors:** Ornampai Japa, Pannawich Siriwechviriya, Khanuengnij Prakhammin

**Affiliations:** 1 Division of Microbiology and Parasitology, School of Medical Sciences, University of Phayao, Thailand; 2 Department of Applied Statistics, Rajamangala University of Technology Isan, Khon Kaen Campus, Thailand

**Keywords:** beef cattle, digenetic trematodes, *Fasciola* spp, *Paramphistomum* spp, Phayao Lake

## Abstract

**Background and Aim:**

Ruminant flukes, including *Fasciola* spp. and *Paramphistomum* spp., are recognized as the significant parasites in livestock worldwide. Cattle infected by these fluke results in slower growth rate and productivity losses contributing to economic losses. In case of *Fasciola* spp., the parasite is considered as an important zoonotic parasite. This study aimed to investigate the prevalence of fluke invasion in beef cattle around Phayao Lake, Phayao, Thailand, between January 2019 and June 2019.

**Materials and Methods:**

A total of 311 fecal samples from beef cows reared nearby Phayao Lake were examined for the presence of fluke eggs by formalin-ethyl acetate sedimentation and subsequently identified by morphology together with methylene blue staining.

**Results:**

The overall prevalence of fluke invasion in beef cattle around Phayao Lake was 33.8% (105/311). The prevalence of rumen fluke and liver fluke was 25.4% (79/311) and 8.4% (26/311), respectively. Mixed infection of both species was found at 1.9% (6/311).

**Conclusion:**

Age of cattle was observed to be associated with invasion rate of all flukes, particularly in the ages over 4 years, which was the highest group of invasion. However, other risk factors, including gender, breed, and location of animals, were not found to be related. This study provides the current status of natural fluke invasion among the beef cattle in Phayao, Thailand, which could be critical for designing the control program of these parasites.

## Introduction

Paramphistomosis and fasciolosis are two economically important parasitic diseases that affect livestock production development in many parts of the world including Thailand [1,2]. The rumen flukes (*Paramphistomum* spp.) and liver flukes (*Fasciola* spp.) are globally distributed and can infect a wide range species of mammalian hosts. Thus, they are responsible for significant financial loss in many countries. Fluke invasions in cattle have negative impacts on the quality and quantity of animal productivity such as reduction in milk yield and growth rate [3,4]. Furthermore, liver fluke has been recognized as the zoonotic fluke infecting both animal and human hosts [5] while the rumen flukes are recently highlighted as an emerging invasive agent of all ruminants [6,7].

Both of rumen and liver flukes are common in many biological characteristics. Their life cycle is similarly complex which requires several hosts for development and shares same intermediate as well as definitive hosts [8]. Infection initiates when definitive host ingests the vegetation or plant containing the metacercariae. Besides, their eggs are very similar in morphological structure which may lead to misdiagnosis and subsequent failure treatment [9-11].

Several studies have described the prevalence of fluke invasion in many ruminant species in Thailand and neighbor countries [2,12-14]. Such data allow us to identify the predominant parasite species affecting livestock and understand the potential associated risk factors of invasion. Moreover, it is essential information for developing effective strategies for prevention and control of parasite invasion.

Phayao Lake is the largest natural reservoir in the northern part of Thailand where wide varieties of plants and animals are presented. Crops and animal husbandry are the main agricultural activity around there. In North Thailand, animal farming is mainly small scale, most beef cows are grazed on the communal area together with other ruminants. Therefore, the transmission of parasite from infected animal species to the environment and other species could be possible. There were no published data of fluke invasion in the cattle in this region.

This study aimed to identify and determine the prevalence of the major fluke invasion in beef cattle reared around Phayao Lake by means of classical sedimentation method.

## Materials and Methods

### Ethical approval

Animal usage protocol of this study was approved by the University of Phayao Animal Ethics Committee, protocol number UP-AE62-02-04-0002.

### Study design and population

This work was a cross-sectional descriptive study which was conducted in the small beef cattle farms within Muang District, Phayao Province, Thailand, from January 2019 to June 2019. Animals were randomly selected from seven villages around Phayao Lake, Phayao, Thailand. A total of 311 ground fecal samples were collected from the beef cattle. Fecal sample collection was carried out between January 2019 and June 2019.

### Study area

Phayao Lake is the largest freshwater reservoir in North Thailand. It is located in Muang District, Phayao Province, which is situated in the northern part of Thailand (19°10’N99°52’E). The lake covered a total area of 19.8 km^2^. The agricultural activities around are crops and animal husbandry. The animals were randomly selected from the small scale farms in seven villages around Phayao Lake. The villages are San Kwan, Toon Tai, Sang, Sang Tai, Morn Kaew, San Pa Kang, and San Wiang Mai.

### Parasitological examination

Approximately 20 g of fecal samples were freshly collected and 3 g of feces was processed for recovery of the parasite eggs by formalin-ethyl acetate sedimentation. Fecal sediments were observed under microscope for the presence of fluke eggs. Methylene blue staining together with egg morphology characterization was employed for differentiation between egg of liver fluke and rumen flukes [11,15].

### Statistical analysis

The prevalence of invasion was estimated from the proportion of positive results to the total number of examined cattle for each parameter. An association between the prevalence of invasion and variable factors, including location, age, sex, and breed of cattle, was analyzed by Chi-square test. The differences among variables were considered statistically significant when p≤0.05.

## Results

### Overall prevalence of fluke infection

For this cross-sectional study, we examined 311 bovine fecal samples collected from small scale farms in seven villages located nearby Phayao Lake between January 2019 and June 2019. Up to 105 of 311 fecal samples were found to be positive for trematode eggs with an overall prevalence of 33.8% (105/311). According to the egg morphology and methylene blue staining characteristics, only two different types of fluke eggs, including *Fasciola* spp. and *Paramphistomum* spp., were observed. The prevalence of rumen fluke invasion was 25.4% (79/311) while the liver fluke invasion was 8.4% (26/311). Mixed invasion between rumen fluke and liver fluke was observed in 6 samples (1.9%). There was no detection of blood or lung fluke eggs at the time of sampling.

Invasion of fluke was found in the cattle from every village examined. The prevalence of fluke invasion in each of the villages ranged from 16.1% to 45.2%. There was no significant difference in the proportions of positive cattle among different villages (p>0.05). The prevalence of overall fluke invasions across seven villages around Phayao Lake is presented in Table-1.

**Table-1:** Prevalence of fluke infection among beef cattle reared nearby Phayao Lake, Phayao, North Thailand (seven villages).

Villages	Number of animals	Number of positive	Prevalence (%)
San Kwan	38	8	21.1
Toon Tai	28	10	35.7
Sang	38	14	36.8
Sang Tai	31	5	16.1
Morn Kaew	115	41	35.7
San Pa Kang	31	14	45.2
San Wiang Mai	30	13	43.3
Total	311	105	33.8

### Age-specific prevalence

Our study, the cattle were grouped according to their ages as follows: <2, 2-4, and over 4 years, the prevalence of fluke invasion was 17.3%, 32.3%, and 55.7%, respectively. The percentage of animals infected with trematode varied in different age groups, ranged from 14.3 to 35.7% and 5.6 to 17.1% for rumen fluke and liver fluke, respectively. The cattle in age over 4 years displayed the most prevalence of infection for rumen fluke (35.7%) and liver fluke (17.1%). There was a significant correlation between invasion rate and age groups in both rumen fluke and liver fluke invasions. In addition, the significant association of age was also observed in mixed invasion of these flukes. Data of sample and prevalence of fluke invasion according to each parameter are summarized in Table-2.

**Table-2:** Prevalence of fluke infection in beef cattle reared around Phayao Lake according to the age, sex, and breed of cattle.

Parameter	Number of animals	Number of infected animals (prevalence) (%)		

		Rumen fluke	Liver fluke	Mixed invasion
Age (years)				
<2	98	14 (14.3)	6 (6.1)	1 (1.0)
2-4	143	40 (28.0)	8 (5.6)	1 (0.7)
>4	70	25 (35.7)	12 (17.1)	4 (5.2)
Sex				
Male	117	31 (26.5)	11 (9.4)	3 (2.6)
Female	194	48 (24.7)	15 (7.7)	3 (1.6)
Breed				
Local	157	34 (21.7)	15 (9.6)	3 (1.9)
Exotic	135	41 (30.4)	10 (7.4)	2 (1.5)
Cross	19	4 (21.1)	1 (5.3)	1 (5.3)
Total	311	79 (25.4)	26 (8.4)	6 (1.9)

### Sex-specific prevalence

The prevalence of overall fluke invasion was slightly higher in male cows (19.3%) than that of female cows (12.5%), however, there was no statistical difference (p>0.05). The proportion of rumen fluke invasion in cattle of both sexes was comparable prevalent at 26.5% and 24.7% for male and female cows, respectively. Similarly, the positivity rate of liver fluke invasion was 9.4% and 7.7% in male and female cattle, respectively. In all cases, the number of infected cattle was non-significantly different among gender (p>0.05).

### Breed-specific prevalence

On breed basis, the prevalence rate of overall fluke invasion was equal proportion at 31.8%, 35.6%, and 36.9% for local, exotic, and crossbreeds, respectively. It was observed that the local breed cattle had a higher prevalence of liver fluke invasion (9.6%) than that of exotic (7.4%) and crossbreeds (5.3%). In contrast, the proportion of rumen fluke invasion was greater in the exotic cattle (30.4%) compared to local breeds (21.7%) and crossbreeds (21.1%). Nevertheless, the difference was non-significant.

## Discussion

The current study revealed that the prevalence of fluke invasion in the beef cattle in North Thailand was 33.8%, which was equal level as previously observed in dairy cattle in Thailand at 32.1% [2]. Parasitic fluke invasion was detected in cattle from every village examined. The highest prevalence of overall trematode invasion was observed in San Pa Kang village (45.2%) and San Wiang Mai village (43.3%) while the lowest rate was found in Sang Tai village (16.1%) and San Kwan village (21.1%). In agreement with the others, rumen and liver flukes are two common flukes infecting cattle and other livestock in Thailand [2,13]. In this study, none of the beef cows had lung or blood fluke invasions. In addition, we observed that rumen fluke was more prevalent (25.4%) than liver fluke (8.4%). This result was consistent with the previous studies from the other parts of Thailand, for instant, northeast, where 44.6% of beef cattle were positive to rumen fluke and 0.5% were positive to liver fluke [16].

In our study, there was no association between fluke invasion and breed of cattle, similar to the findings of the previous studies [17,18]. Conversely, Martinez-Ibeas *et al*. [19] reported the significant differences in ovine breed positivity with rumen fluke invasion. However, significant differences were not observed among the breed of cows in the present study (p>0.05).

In agreement with other studies, we found that there was no association between sex of animals and prevalence of either rumen or liver flukes invasion. A similar observation was recorded by Magaji *et al*. [17] and Shinggu *et al*. [20] who reported an equal proportion of infected female and male cattle. Many research groups reported that there was no significant difference in the prevalence of infected male and female cattle. As a result, it could be assumed that both sexes of cattle have the same chance of being infected when they are exposed to similar risk conditions of invasion.

Our results indicated that the age of animals had influence on the proportion of cattle infected with either single invasion of rumen or liver fluke. The invasion rate was increased with the age of tested cattle. In addition, the significant association of age was also observed in case of mixed invasion of these two flukes. These findings were consistent with previous observation in native cattle and crossbreed cattle from Bangladesh, in which the highest prevalence of the above parasites was observed in animals aged over 3 years [21]. It might be speculated that the older cattle are more likely to prolonged exposure time to infective stage of a fluke than those of younger animals.

Animals being infected with the fluke are considered to be the sources of releasing the parasite’s egg to the environment before transmitting to the snail intermediate host. However, several factors are believed that contributed to the parasitic fluke distribution in each area, the presence of related intermediate hosts along with the favor environmental conditions, for instance, season, humidity, and temperature could be the key factors influenced the successive completion of parasite life cycle [8].

## Conclusion

This work provides the current data of trematode invasion among beef cattle around Phayao Lake. Obviously, rumen flukes and liver flukes were major parasitic flukes of the beef cattle in this area. Our finding indicated that rumen fluke was more prevalent than liver fluke. In addition, coinfection of both flukes was also recorded. We suggested that the age of animal was the general risk factor associated with the presence of rumen and liver fluke infection in the studied beef cattle while location, gender, and breed of animals were not found to be associated.

An occurrence of these two parasites implies that it could contribute to the opportunity of the infective stage of parasites spreading and contaminating in the area. Consequently, the other species of ruminants and human hosts could be at risk of obtaining infective stage of these parasites. This epidemiological data could be critical for encouraging the development of the prevention and control scheme to minimize the above parasite in this area.

## Authors’ Contributions

OJ and KP designed the study and analyzed the data. OJ and PS performed the experiments. OJ drafted and revised the manuscript. All authors read and approved the final manuscript.
